# Psychometric properties of the hospital survey on patient safety culture, HSOPSC, applied on a large Swedish health care sample

**DOI:** 10.1186/1472-6963-13-332

**Published:** 2013-08-22

**Authors:** Mats Hedsköld, Karin Pukk-Härenstam, Elisabeth Berg, Marion Lindh, Michael Soop, John Øvretveit, Magna Andreen Sachs

**Affiliations:** 1 Medical Management Centre, Karolinska Institutet, SE 171 77 Stockholm, Sweden; 2 Astrid Lindgren´s Childrens´ Hospital, Karolinska University Hospital, SE 171 76 Stockholm, Sweden; 3 Public Healthcare Services Committee Administration, Stockholm County Council, Box 6909, SE 102 39 Stockholm, Sweden; 4 National Board of Health and Welfare, SE 106 30 Stockholm, Sweden

**Keywords:** Patient safety culture, Hospital Survey on Patient Safety Culture, S-Hospital Survey on Patient Safety Culture, Primary care, Psychometric properties, Construct validity, Internal consistency

## Abstract

**Background:**

A Swedish version of the USA Agency for Healthcare Research and Quality “Hospital Survey on Patient Safety Culture” (S-HSOPSC) was developed to be used in both hospitals and primary care. Two new dimensions with two and four questions each were added as well as one outcome measure. This paper describes this Swedish version and an assessment of its psychometric properties which were tested on a large sample of responses from personnel in both hospital and primary care.

**Methods:**

The questionnaire was mainly administered in web form and 84215 forms were returned (response rate 60%) between 2009 and 2011. Eleven per cent of the responses came from primary care workers and 46% from hospital care workers. The psychometric properties were analyzed using both the total sample and the hospital and primary care subsamples by assessment of construct validity and internal consistency. Construct validity was assessed by confirmatory (CFA) and exploratory factor (EFA) analyses and internal consistency was established by Cronbachs’s α.

**Results:**

CFA of the total, hospital and primary care samples generally showed a good fit while the EFA pointed towards a 9-factor model in all samples instead of the 14-dimension S-HSOPSC instrument. Internal consistency was acceptable with Cronbach’s α values above 0.7 in a major part of the dimensions.

**Conclusions:**

The S-HSOPSC, consisting of 14 dimensions, 48 items and 3 single-item outcome measures, is used both in hospitals and in primary care settings in Sweden for different purposes. This version of the original American instrument has acceptable construct validity and internal consistency when tested on large datasets of first-time responders from both hospitals and primary care centres. One common instrument for measurements of patient safety culture in both hospitals and primary care settings is an advantage since it enables comparisons between sectors and assessments of national patient safety improvement programs. Future research into this version of the instrument includes comparing results from patient safety culture measurements with other outcomes in relation to safety improvement strategies.

## Background

Assessments of safety culture are made in many industries outside of healthcare. In some studies, an association between high safety culture scores and unsafe behavior and accidents has been found [1,2].

In health care, safety culture assessments have been made for almost a decade in the US [3,4]. A growing number of studies report on their value and use, both in the US and internationally [5,6]. A recent study suggested that improvements in clinical outcomes correlated positively with improvement in safety culture as measured by Safety Attitude Questionnaire [7]. However, researchers have noted the need for more standardized use of terms and a greater understanding of how safety culture as measured is related to other features of healthcare as well as the need to develop theoretical models to explain the influence of culture on patient safety outcomes [8,9].

The Agency for Health Care Research and Quality (AHRQ) “Hospital Survey on Patient Safety Culture” (HSOPSC) is one questionnaire instrument commonly used in the USA [3] and increasingly used internationally, with and without modifications. There is a growing body of literature on the HSOPSC and other tools such as the Safety Attitude Questionnaire for measuring safety culture [10-12]. A lack of knowledge about the validity of the factor structure of versions of these instruments has been noted which may limit their use and usefulness [13]. Erbes et al. 2004 proposed that an important consideration for evaluating a measure is the independence of the “factors” which structure the instrument–that the different dimensions are relatively independent [14].

There have been studies which have validated the factor structure of some of the non-US HSOPSC-based instruments: a study of the Japanese version was shown to have a good fit with the factor structure of the original instrument [15]. In a UK study, confirmatory factor analysis, however, showed a weak fit calling for a slight remodelling of the factor structure [16]. Also, in three other European studies, confirmatory factor analysis did not fully replicate the structure of the original instrument [5,13,17]. One weakness with some of the above mentioned studies is the relatively small sample sizes used for the factor analyses since that may influence the result of these analyses. Nevertheless, these findings have raised questions about the applicability of the US HSOPSC in other countries and whether an instrument for patient safety culture measurements can be exported across national borders and health care systems [16]. It is possible that there are significant differences between health care environments which weaken the validity and usefulness of the instrument. These findings suggest that this and other safety culture or safety climate instruments require careful testing before being widely used or before drawing conclusions about their meaning in countries or contexts other than those for which they were developed.

The purpose of this study was further to investigate certain psychometric properties of the Swedish version of HSOPSC in order to contribute to knowledge about international examples of the HSOPSC and to guide its use within Sweden.

### Why was HSOPSC chosen for a Swedish sample?

In 2007 a national network of safety practitioners and researchers in Sweden concluded that measurement of safety culture could contribute to improvement and understanding of patient safety. The Hospital Survey on Patient Safety Culture (HSOPSC) was recommended by Medical Management Centre at Karolinska Institutet because this instrument had undergone extensive development and testing, was widely used in the US and because comparisons with the US could be informative. This recommendation was also supported by a study describing the AHRQ instrument as the only patient safety culture instrument which was based on a comprehensive scale development [18]. The US HSOPSC instrument includes 12 safety dimensions and 42 items, as well as two single-item outcome questions and additional background questions. An exploratory factor analysis had been performed to explore the dimensionality of the HSOPSC [19]. This study was later repeated on a larger dataset with confirmatory results [20]. Also, the European Society for Quality in Healthcare in a project funded by the European Commission has recommended the HSOPSC as one of three instruments for measuring patient safety culture in European countries [12].

When the instrument was chosen for use in Sweden it was regarded likely that it would be suitable both for hospitals and primary care centres. The Swedish health care system is a tax based public system organised as 21 geographical county health systems which are responsible for both primary and hospital care.

Two years after the instrument had been introduced in Swedish healthcare it became a governmental requirement, linked to reimbursement, for health care organisations to measure patient safety culture and to issue reports on improvement strategies [21]. The Swedish version of the instrument (S-HSOPSC) is now used by all county councils and findings from the surveys are published in annual reports by the Swedish Association of local Authorities and Regions and the National Board of Health and Welfare. The psychometric properties of this version of the instrument (S-HSOPSC) have not until now been assessed.

The aim of this paper is to describe the S-HSOPSC for use in hospitals and in primary care settings, report the results of examining its psychometric properties on a large sample of responses and provide recommendations for further development in Sweden and elsewhere.

## Methods

### The Swedish version of the HSOPSC

The questionnaire was translated into Swedish by a professional translator. The translation was checked by four health care and patient safety experts to ensure correct terminology and was then back-translated by another translator and minor discrepancies between the versions were solved by the experts and the translator in collaboration. The other key differences from the US AHRQ instrument are, so as to meet the Swedish Patient Safety Act [22], the addition of

• one “outcome” question about the number of risk reports submitted (17/G2)

• four questions about “Information and support to patients and family who have suffered an adverse event”, (the dimension 13, items G3, G4, G5, G6).

Further, two questions about “Information and support to staff who have been involved in an adverse event” (Dimension 14, items G7, G8) were also added to the S-HSOPSC.

These additional questions were formulated by three of the authors (ML, MS, MAS) using the wordings in the Act (G3, G4, G5, G6) and applying the same type of wordings for the questions about staff information and support. Finally, since the instrument was meant to be used not only in hospitals but also in primary care settings, the word hospital was either omitted or exchanged for a more generic term (e.g. unit or organisation).

The Swedish version of the instrument thus has 14 dimensions, 48 items and three single-item “outcome” questions (15/E, 16/G1 and17/G2), as shown in Table 1.

**Table 1:** Dimensions and items of the S-HSOPSC

**1**	**Communication openness**
C2	Staff will freely speak up if they see something that may negatively affect patient care
C4	Staff feel free to question the decisions or actions of those with more authority
C6r	Staff are afraid to ask questions when something does not seem right
**2**	**Feedback and communication about error**
C1	We are given feedback about changes put into place based on event reports
C3	We are informed about errors that happen in this unit
C5	In this unit, we discuss ways to prevent errors from happening again
**3**	**Frequency of error reporting**
D1	When a mistake is made, but is caught and corrected before affecting the patient, how often is this reported?
D2	When a mistake is made, but has no potential to harm the patient, how often is this reported?
D3	When a mistake is made that could harm the patient, but does not, how often is this reported?
**4**	**Handoffs and transitions between units and shifts**
F3r	Things “fall between the cracks” when transferring patients from one unit to another
F5r	Important patient care information is often lost during shift changes
F7	Problems often occur in the exchange of information across units
F11	Shift changes are problematic for patients in this unit
**5**	**Executive management support for patient safety**
F1	Executive management provides a work climate that promotes patient safety
F8	The actions of executive management show that patient safety is a top priority
F9	Executive management seems interested in patient safety only after an adverse event happens
**6**	**Nonpunitive response to error**
A8	Staff feel like their mistakes are held against them
A12	When an event is reported, it feels like the person is being written up, not the problem
A16	Staff worry that mistakes they make are kept in their personnel file
**7**	**Organizational learning–continuous improvement**
A6	We are actively doing things to improve patient safety
A9	Mistakes have led to positive changes here
A13	After we make changes to improve patient safety, we evaluate their effectiveness
**8**	**Overall perceptions of safety**
A15	Patient safety is never sacrificed to get more work done
A18	Our procedures and systems are good at preventing errors from happening
A10	It is just by chance that more serious mistakes don´t happen around here
A17	We have patient safety problems in this unit
**9**	**Staffing**
A2	We have enough staff to handle the workload
A5	Staff in this unit work longer hours (scheduled hours including overtime) than is best for patient care
A7	We use more agency/temporary staff than is best for patient care
A14	We work in “crisis mode”, trying to do too much, too quickly
**10**	**Supervisor/manager expectations and actions promoting safety**
B1	My supervisor/manager says a good word when he/she sees a job done according to established safety procedures.
B2	My supervisor/manager seriously considers staff suggestions for improving patient safety
B3	Whenever pressure builds up, my supervisor/manager wants us to work faster, even if it means taking shortcuts
B4	My supervisor/manager overlooks patient safety problems that happen over and over
**11**	**Teamwork across units**
F4	There is good cooperation among units that need to work together
F10	Units work well together to provide the best care for patients
F2	Units do not coordinate well with each other
F6	It is often unpleasant to work with staff from other units
**12**	**Teamwork within the unit**
A1	People support one another in this unit
A3	When a lot of work needs to be done quickly, we work together as a team to get the work done
A4	In this unit, people treat each other with respect
A11	When one area in this unit gets really busy, others help out
**13**	**Information and support to patients and family who have suffered an adverse event**
G3	In this unit, apologies and regrets are given to patients and families who have suffered an adverse event
G4	In this unit, patients and families who have suffered an adverse event are informed about the event, its causes and actions taken to prevent it from happening again
G5	In this unit, patients and families who have suffered an adverse event, receive help and support in order to manage the situation
G6	In this unit, patients and families who have suffered an adverse event, are informed about the possibility to apply for economic compensation from the Patient Insurance
**14**	**Information and support to staff who have been involved in an adverse event**
G7	In this unit, staff who have been involved in an adverse event, receive information about actions taken to prevent the event from happening again
G8	In our unit, staff who have been involved in an adverse event, receive help and support in order to manage the situation
**15**	**Patient safety grade**
E	Please give your unit an overall grade on patient safety
**16**	**Number of events reported**
G1	In the past 12 months, how many event reports have you filled out and submitted?
**17**	**Number of risks reported**
G2	In the past 12 months, how many risk reports have you filled out and submitted?

### Ethical considerations

Ethics approval was obtained from the Regional Ethics Committee of Stockholm (Number 2010/820-31/5).

### Cognitive testing and first pilot testing

Focus groups were used for initial cognitive testing of the S-HSOPSC which involved asking staff about how they perceived the questions. No changes were needed. A pilot testing was then carried out by letting a group of doctors and nurses working in primary and hospital care (n = 78) fill out the questionnaire. The participants also answered questions on their reactions to and thoughts about the instrument. Minor amendments were made and the final version was approved by a second focus group without further remarks.

### Further pilot testing

A validity assessment of the factor structure of the S-HSOPSC was made by distributing questionnaires by mail or web based to all staff in primary care centres and hospital departments that had volunteered to participate in a pilot testing of the instrument as part of their strategic, long-term patient safety programs in 2008 (n = 3114). Response rate in this pilot survey was 56%. About half of the returned questionnaires had all items answered, i.e. could be used for the statistical analyses. The primary care sample, thus suitable for statistical analysis, turned out to be too small for assessments of psychometric properties.

Due to the spread and increased use of the instrument, mainly because of the governmental requirement to measure patient safety culture, the database of returned questionnaires grew substantially. At the beginning of 2012 the research group received permission from the owners of the material (the county councils) to use the database for the purpose of testing of construct validity and internal consistency.

### Sample properties and response rates

**Figure 1:**
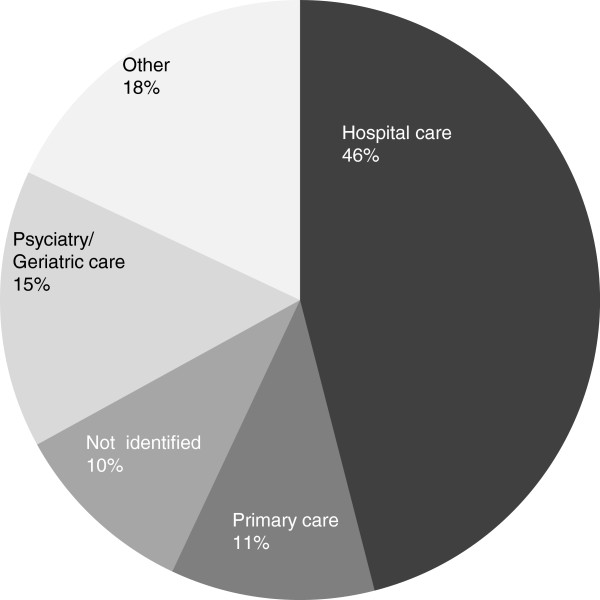
Respondents’ work area.

**Figure 2:**
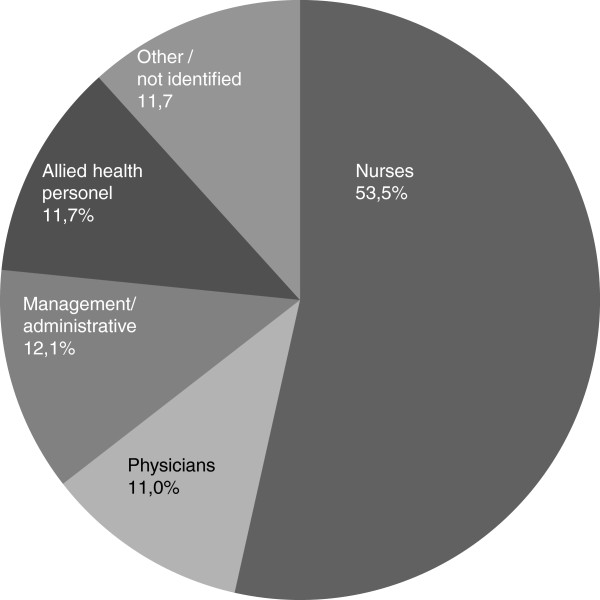
Respondents’ profession.

The national data base includes 84 215 questionnaires (response rate 60%), returned between 2009 and 2011 (all first-time responders), and this data base was used in this study. Less than 6% of the responses in this data base are paper based questionnaires, the rest is web based. All county councils except one have provided data to the database and all returned questionnaires are the first measurements of safety culture using this instrument. The county councils use the survey as part of their strategic patient safety improvement work and decisions about which organizations should be included in the survey were made by them. The authors have had no influence on who received the questionnaire. Forty six per cent of the responders represent different types of hospitals including university, larger regional and smaller rural hospitals and 11% of responders represent primary care centres. The work area for the respondents in this data base is shown in Figure 1 and profession in Figure 2. For further analysis, two subsets of the total database were extracted for analyses: hospital and primary care samples.

### Statistical analyses

For the statistical analyses only returned questionnaires with all items answered were used. Since the number with all items answered was large both for the complete sample and the two sample subsets there was no need to replace missing values. We calculated the Kaiser-Meyer-Olkin measure of sample adequacy (KMO) to establish the adequacy of the sample for factor analysis [23].

#### Construct validity

The analysis of the construct validity, i.e. assessing the links between items and relations between items and an underlying dimension, was made by performing confirmatory factor analyses (CFA) to determine the degree of fit between our sample and a hypothesized measurement model [24]. The following fit measures were used: Comparative Fit Index (CFI), Goodness of Fit index (GFI), Adjusted Goodness of Fit index (AGFI), Normalized Fit Index (NFI) and Non-normalized Fit Index (also known as Tucker-Lewis Index) (NNFI). These measures range from 0 (poor fit) to 1 (perfect fit) and 0.9 was chosen as acceptable level of fit [25]. The measure Root Mean Square Error of Approximation (RMSEA) (limit for acceptable fit: below 0.05) was also applied. Construct validity of the S-HSOPSC was further assessed by variance tests between items using Standardized path coefficient (limit ≥0.5) and Squared multiple correlations (ItemR^2^) (limit ≥0.3). The proportion of common item variance, i.e. communalities, was calculated in order to detect common underlying dimensions (limit ≥0.4) [23]. Based on these results average variance extracted (AVE) and construct reliability (CR) for each factor were calculated in order to determine convergent validity. Acceptable values for AVE were: ≥ 0.5 and for CR: > AVE [26].

To further assess the construct validity, an exploratory factor analysis (EFA) was performed. Different techniques are available. Due to the nature of the data material where correlations between factors are allowed we chose an oblique rotation method using Promax which is a procedure designed for very large datasets [23]. Furthermore, factor analysis (principal axis factor analysis, PAF) was used to identify factors and correlations among measured items [27]. Level for acceptable factor loading was set at ≥ 0.4 [23]. Based on the EFA, the residual correlation matrix was also calculated, i.e. the differences between the observed correlation coefficients and the correlations estimated from the model (should be <0.05) [23].

Finally, correlations between the dimensions 1–14 and the outcome questions were studied by the non-parametric Spearman-Rho correlation (0.0–0.25 little or no relationship; 0.25–0.50 fair degree of relationship; 0.50–0.75 moderate to good relationship; >0.75 very good to excellent relationship) [28].

#### Internal consistency

Internal consistency was established by Cronbach’s α (criterion: ≥0.7 for each dimension) [25]. Cronbach’s α tests were performed separately on the complete sample, the hospital and the primary care samples where all items within each dimension under study had been answered.

Statistical analyses were performed using SPSS 19 and AMOS 19.

## Results

### Response rates

The total number of returned questionnaires and the number of questionnaires with all items answered are shown in Table 2.

**Table 2:** Number of returned questionnaires with all items answered

**Sample**	**Returned questionnaires**	**All items answered**
Total sample	84215	39396
Hospital care sample	38812	21099
Primary care sample	9113	3518

Generally, response rates per item were satisfactory. Lowest values were 74% (total sample) and 78% (hospital sample) for item G6 (“*In this unit, patients and families who have suffered an adverse event, are informed about the possibility to apply for economic compensation from the Patient Insurance”*) and 60% (primary care sample) for item F11 (“*Shift changes are problematic for patients in this unit”*).

### Construct validity

KMO was 0.95 for all three samples confirming the adequacy for factor analysis. CFA of the complete sample and the hospital and the primary care samples generally showed a good fit for our Swedish 14 dimension instrument. Only AGFI was slightly below the set margin 0.9 for the primary care sample (Table 3).

**Table 3:** Summary of fit indexes

	**Complete sample**	**Hospital care sample**	**Primary care sample**
The comparative fit index (CFI)	0.91	0.91	0.91
The goodness of fit index (GFI)	0.92	0.92	0.90
The adjusted GFI (AGFI)	0.90	0.91	0.89
The normalized fit index (NFI)	0.91	0.91	0.90
The non-normalized fit index (NNFI)	0.90	0.90	0.90
Root Mean Square Error of Approximation (RMSEA)	0.042	0.042	0.042

Acceptable level of fit for CFI, GFI, AGFI, NFI and NNFI ≥0.9 and for RMSEA ≤0.05.

Further testing for construct validity by variance tests revealed that five items were below the 0.3 limit in Item R^2^ (A5, A7, A15, F6 and F11) of which items A15 (“*Patient safety is never sacrificed to get more work done”*) and A7 (“*We use more agency/temporary staff than is best for patient care”*) had less than 20% of their variability explained by the model in all samples. These two items also dropped below the 0.5 cut off in standardized path coefficient calculations in all samples. Communality values were below the 0.4 level for 13 items in the total sample, and10 and 8 items in the hospital and primary care samples, respectively. Among these, those with the lowest values (< 0.2) were A7 and A15 (Table 4).

**Table 4:** Summary of variance testing

**Dimension**	**Item**	**Item R**^**2**^			**Std path coefficient**			**Communalities**		
		**Total sample**	**Hospital care sample**	**Primary care sample**	**Total sample**	**Hospital care sample**	**Primary care sample**	**Total sample**	**Hospital care sample**	**Primary care sample**
1	C2	0.45	0.45	0.47	0.67	0.67	0.68	0.39	0.38	0.42
	C4	0.39	0.37	0.42	0.62	0.61	0.65	0.30	0.28	0.36
	C6r	0.42	0.39	0.45	0.64	0.63	0.67	0.38	0.35	0.43
2	C1	0.41	0.41	0.48	0.64	0.64	0.69	0.43	0.43	0.46
	C3	0.48	0.48	0.55	0.70	0.70	0.74	0.51	0.51	0.50
	C5	0.65	0.64	0.67	0.81	0.80	0.82	0.61	0.60	0.66
3	D1	0.73	0.73	0.77	0.86	0.85	0.88	0.72	0.71	0.77
	D2	0.75	0.74	0.78	0.87	0.86	0.88	0.79	0.78	0.81
	D3	0.59	0.58	0.62	0.77	0.76	0.79	0.58	0.57	0.61
4	F3r	0.49	0.44	0.54	0.70	0.67	0.73	0.50	0.49	0.51
	F5r	0.39	0.43	0.36	0.62	0.66	0.60	0.48	0.47	0.55
	F7r	0.53	0.51	0.53	0.73	0.72	0.73	0.53	0.53	0.50
	F11r	0.26	0.32	0.21	0.51	0.57	0.46	0.39	0.41	0.39
5	F1	0.66	0.68	0.61	0.81	0.82	0.78	0.60	0.62	0.55
	F8	0.70	0.71	0.63	0,83	0.84	0.79	0.63	0.64	0.56
	F9r	0.42	0.41	0.41	0.65	0.64	0.64	0.41	0.41	0.37
6	A8r	0.54	0.55	0.53	0.74	0.74	0.73	0.48	0.50	0.49
	A12r	0.55	0.56	0.57	0.74	0.75	0.75	0.50	0.52	0.51
	A16r	0.41	0.40	0.41	0.64	0.63	0.64	0.43	0.42	0.43
7	A6	0.53	0.54	0.55	0.73	0.73	0.74	0.46	0.47	0.46
	A9r	0.30	0.32	0.29	0.54	0.56	0.54	0.28	0.30	0.27
	A13	0.42	0.42	0.43	0.64	0.65	0.66	0.38	0.38	0.40
8	A15	0.19	0.19	0.16	0.43	0.44	0.41	0.19	0.20	0.16
	A18	0.38	0.40	0.37	0.62	0.63	0.61	0.39	0.41	0.38
	A10r	0.53	0.54	0.48	0.73	0.73	0.69	0.51	0.53	0.48
	A17r	0.58	0.57	0.56	0.76	0.76	0.75	0.51	0.52	0.48
9	A2	0.48	0.52	0.50	0.69	0.72	0.71	0.45	0.46	0.46
	A5r	0.25	0.24	0.21	0.50	0.49	0.45	0.26	0.24	0.23
	A7r	0.15	0.15	0.14	0.39	0.39	0.37	0.18	0.18	0.18
	A14r	0.59	0.57	0.61	0.77	0.75	0.78	0.52	0.51	0.53
10	B1	0.47	0.47	0.51	0.69	0.69	0.71	0.52	0.51	0.55
	B2	0.68	0.69	0.66	0.82	0.83	0.82	0.72	0.73	0.66
	B3r	0.33	0.35	0.32	0.58	0.59	0.57	0.39	0.42	0.40
	B4r	0.52	0.53	0.49	0.72	0.73	0.70	0.53	0.55	0.50
11	F4	0.49	0.48	0.48	0.70	0.69	0.70	0.44	0.41	0.48
	F10	0.48	0.48	0.46	0.69	0.69	0.68	0.44	0.43	0.47
	F2r	0.45	0.45	0.44	0.67	0.67	0.66	0.45	0.45	0.44
	F6r	0.23	0.20	0.30	0.51	0.49	0.55	0.28	0.24	0.41
12	A1	0.56	0.54	0.55	0.75	0.73	0.74	0.55	0.56	0.55
	A3	0.43	0.43	0.52	0.65	0.66	0.72	0.49	0.45	0.57
	A4	0.54	0.52	0.56	0.73	0.72	0.75	0.52	0.53	0.53
	A11	0.31	0.31	0.43	0.56	0.56	0.66	0.34	0.32	0.46
13	G3	0.54	0.53	0.55	0.73	0.73	0.74	0.50	0.50	0.51
	G4	0.70	0.69	0.72	0.84	0.83	0.85	0.68	0.67	0.69
	G5	0.58	0.59	0.60	0.76	0.77	0.77	0.61	0.62	0.63
	G6	0.41	0.42	0.46	0.64	0.65	0.68	0.42	0.43	0.46
14	G7	0.68	0.68	0.71	0.82	0.82	0.68	0.54	0.53	0.60
	G8	0.59	0.59	0.65	0.77	0.77	0.81	0.47	0.46	0.56

Limits for standardized path coefficient ≥0.5 and for Item R^2^ ≥0.3.

AVE showed values below the 0.5 level for almost half of all dimensions with lowest values for dimension 8 “*Overall perceptions of safety”* and 9 “*Staffing”* in all samples. CR values, however, were above the AVE values in all dimensions (Table 5).

**Table 5:** Summary of average variance extracted (AVE) and construct reliability (CR)

	**Total sample**		**Hospital care sample**		**Primary care sample**	
**Dimension**	**AVE**	**CR**	**AVE**	**CR**	**AVE**	**CR**
1	0.42	0.68	0.40	0.67	0.44	0.71
2	0.51	0.76	0.51	0.76	0.57	0.80
3	0.69	0.87	0.68	0.87	0.72	0.89
4	0.42	0.60	0.43	0.62	0.41	0.57
5	0.59	0.81	0.60	0.81	0.55	0.78
6	0.50	0.75	0.50	0.75	0.50	0.75
7	0.41	0.68	0.42	0.69	0.43	0.69
8	0.37	0.74	0.38	0.75	0.35	0.73
9	0.37	0.52	0.37	0.51	0.36	0.50
10	0.50	0.69	0.51	0.70	0.50	0.68
11	0.41	0.59	0.40	0.58	0.42	0.60
12	0.46	0.63	0.45	0.63	0.52	0.70
13	0.56	0.74	0.56	0.74	0.58	0.76
14	0.63	0.87	0.64	0.87	0.68	0.89

Acceptable levels for AVE ≥0.5 and for CR ≥ AVE.

Results of EFA by using principal axis factoring (PAF) as extraction method and Promax as rotation method are presented in Additional file 1: Appendix 1. The EFA indicated 9 factors in all three samples in contrast to the 14 dimensions of the instrument. The factors jointly explained 56.4% of the total variance of all the items. Dimension 7 *“Organizational learning–continuous improvement”* and dimension 2 *“Feedback and communication about error”* and two of three items from dimension 1 *“Communication openness*” as well as one item from dimension 8 *“Overall perceptions of safety*” all loaded onto factor 1. The remaining items from dimension 8 loaded onto dimension 9 *“Staffing”*. The new Swedish dimensions 13 “*Information and support to patients and family who had suffered an adverse event”* and 14 “*Information and support to staff who have been involved in an adverse event”* loaded together as did dimension 5 *“Executive management support for patient safety”* and dimension 11 *“Teamwork across units”.*

Four items showed overall factor loading below 0.4, and so did one more item in the hospital care sample and additional two more items in the total sample. For all three samples these were C4 *“Staff feel free to question the decisions or actions of those with more authority”,* C6 *“Staff are afraid to ask questions when something does not seem right”,* A15 *“Patient safety is never sacrificed to get more work done”* and A7 *“We use more agency/temporary staff than is best for patient care”*. In addition, in both total and hospital samples A18 *“Our procedures and systems are good at preventing errors from happening”* and finally in the total sample F10 *“Units work well together to provide the best care for patients”* were below 0.4. Three items in all samples loaded onto another factor than the S-HSOPSC dimensions (1/C6, 8/A18 and 11/F6) and in the primary care sample two more items in dimension 4 (4/F5 and 4/F11). Nine items from four dimensions loaded onto factor 1 and 7 items from two dimensions loaded onto factor 2 (Additional file 1: Appendix 1).

The non-redundant residuals were 25 (2.0%) with an absolute value above 0.05. The Spearman-Rho correlation, based on the total sample, revealed a fair to good degree of relationship between most of the dimensions and also with the outcome question 15/E. There was no correlation between the two other outcome questions *“Number of events reported”* (16/G1) and *“Number of risks reported”* (17/G2) and the dimensions 1–14 (Table 6).

**Table 6:** Correlation between dimensions and single-outcome questions by non-parametric Spearman-Rho method

	**Dim1**	**Dim2**	**Dim3**	**Dim4**	**Dim5**	**Dim6**	**Dim7**	**Dim8**	**Dim9**	**Dim10**	**Dim11**	**Dim12**	**Dim13**	**Dim14**	**E1**	**G1**
Dim2	.533**															
Dim3	.331**	.446**														
Dim4	.329**	.323**	.253**													
Dim5	.318**	.380**	.315**	.376**												
Dim6	.463**	.384**	.242**	.334**	.329**											
Dim7	.422**	.567**	.404**	.304**	.429**	.348**										
Dim8	.432**	.429**	.339**	.397**	.475**	.484**	.480**									
Dim9	.304**	.274**	.164**	.309**	.392**	.441**	.281**	.536**								
Dim10	.499**	.522**	.332**	.323**	.391**	.463**	.492**	.485**	.387**							
Dim11	.331**	.344**	.268**	.605**	.512**	.316**	.358**	.421**	.315**	.342**						
Dim12	.465**	.424**	.276**	.338**	.278**	.412**	.452**	.422**	.339**	.431**	.344**					
Dim13	.346**	.379**	.339**	.268**	.312**	.243**	.407**	.333**	.186**	.343**	.311**	.308**				
Dim14	.377**	.465**	.364**	.273**	.363**	.289**	.455**	.372**	.229**	.388**	.327**	.322**	.754**			
E	.417**	.455**	.400**	.364**	.463**	.384**	.487**	.659**	.436**	.462**	.404**	.412**	.345**	.381**		
G1	.023**	.070**	.060**	-.030**	-.070**	.047**	.030**	-.084**	-.055**	-.005	-.058**	.023**	-.065**	-.023**	.080**	
G2	.024**	.061**	.102**	-.015**	-.037**	.019**	.063**	-.081**	-.058**	-.001	-.030**	.020**	.014**	.031**	.069**	.387**

**Correlation is significant at the 0.01 level (2-tailed).

0.0–0.25 little or no relationship; 0.25–0.50 fair degree of relationship; 0.50–0.75 moderate to good relationship; >0.75 very good to excellent relationship.

### Internal consistency

Results of internal consistency analysis are presented in Table 7. Of the 14 groupings of items into dimensions, 2 dimensions, i.e. dimensions 7 “*Organizational learning*–c*ontinuous improvement”* and 9 “*Staffing”* in all samples and dimension 1 “*Communication openness”* in the total and hospital care samples fell short of an adequate level of internal consistency, i.e. were below 0.7.

**Table 7:** Internal consistency by test of Cronbach’s α

	**Dimension**	**Total sample**	**Hospital care sample**	**Primary care sample**
1	Communication openness	0.68	0.67	0.70
2	Feedback and communication about error	0.76	0.76	0.80
3	Frequency of error reporting	0.87	0.87	0.88
4	Handoffs and transitions between units and shifts	0.74	0.75	0.73
5	Executive management support for patient safety	0.81	0.81	0.79
6	Nonpunitive response to error	0.74	0.74	0.75
7	Organizational learning–continuous improvement	0.66	0.66	0.68
8	Overall perceptions of safety	0.71	0.72	0.69
9	Staffing	0.67	0.67	0.64
10	Supervisor/manager expectations and actions promoting safety	0.78	0.79	0.79
11	Teamwork across units	0.72	0.71	0.74
12	Teamwork within the unit	0.76	0.75	0.80
13	Information and support to patients and family who have suffered an adverse event	0.83	0.83	0.84
14	Information and support to staff who have been involved in an adverse event	0.77	0.77	0.81

Criterion ≥0.7 for each dimension.

## Discussion

In this study on psychometric properties of the Swedish version of the AHRQ-instrument (S-HSOPSC) for measurement of patient safety culture, a database containing over 80 000 questionnaires from all sectors of Swedish health care, was used. To our knowledge, a database of a similar size has only been used by Sorra and Dyer in their 2010 examination of the multilevel psychometric properties of the original instrument issued in 2004 [20]. In our study, psychometric tests were performed on the total sample and on two subsamples: the hospital and primary care samples.

The exploratory factor analysis pointed towards a 9-factor model for the total sample and both subsamples in contrast with the 14 dimensions of the instrument. However, confirmatory factor analysis generally showed a good fit between our data in all samples and the 14 dimension instrument with only 2 items, i.e. “*We use more agency/temporary staff than is best for patient care”* and “*Patient safety is never sacrificed to get more work done”* having less than 20% of their variability explained by the model. Also, there was satisfactory convergent validity and a fair to good degree of relationship between all dimensions and the single-item outcome measure *“Patient safety grade*”.

Internal consistency was generally good in all samples with lowest Cronbach´s α values for “*Communication openness”* (total and hospital sample), “*Organizational learning–continuous improvement”* (all samples) and “*Staffing”* (all samples).

Other researchers have reached similar results: low factor loadings for items “*We use more agency/temporary staff than is best for patient care”*[16] and “*Patient safety is never sacrificed to get more work done”*[17]. The latter item was excluded from the Dutch version of the instrument [17]. In contrast with our results, the confirmatory factor analysis carried out on a UK sample by Waterson et al. [16] showed a poor fit and an optimal nine dimension-model was constructed instead [16]. In other studies weak internal consistency has also been demonstrated for the same dimensions as in our study: “*Organizational learning–continuous improvement”*[13,16,17], and “*Staffing”*[13,16,17] Further studies are needed to investigate the possible linkage between certain dimensions and items.

At present, our opinion is that these items and dimensions should be kept since they signify important aspects of patient safety and as such form a useful foundation for improvement work.

Overall, the psychometric properties of the S-HSOPSC proved satisfactory and there is solid evidence for the 14 dimensions and 48 items of the S-HSOPSC. Thus, at present no changes will be made to the instrument. Also, there is a general wish in Swedish health care to keep the instrument as close as possible to the original AHRQ version. These decisions will be reconsidered when repeating the analysis of the psychometric properties of the S-HSOPSC on a dataset of second-time respondents which is now accumulating. The reason for repeating the analysis is that when the database used in this study was collected, the patient safety movement in Sweden was in its beginning and the general awareness of basic concepts of patient safety was probably low for many of the respondents of this questionnaire. This may have affected how the questions were understood and answered.

The instrument was originally designed for hospitals but with few changes of wordings it is also in use within primary care in Sweden. To our knowledge, the use of the AHRQ instrument in primary care has only been reported in Turkey [29] and The Netherlands [30]. Some aspects of patient safety may not be as relevant for some primary care offices as for hospital units, such as questions about handoffs, teamwork across units and executive management support. Because of the absence of a possibility to give a “not applicable” reply, such items might be left unanswered which in turn renders a lower response rate for these items as was the case with the item “*Shift changes are problematic for patients in this unit”* in our primary care sample. Another drawback with modification of words, so as to suit both sectors of the system, may be the risk of a decrease in accuracy of the question. The S-HSOPSC form starts with explanations regarding the interpretation of certain of the words used in the survey to minimize this risk.

There are great advantages with one common instrument for patient safety culture measurements in both the hospital and the primary care sector. Not only does it simplify measurements within the health care system which in Sweden includes primary health care–the entrance to the system–and hospital care but it also provides opportunities for comparisons and learning within the system and assessments of national programs for quality and patient safety improvement. On the other hand, by adapting an instrument designed for hospital care for use in primary care, important aspects on patient safety in this sector of the health care system, might not be captured. This remains to be further studied.

Also, the criterion related validity of the S-HSOPSC needs to be explored by comparing the results from safety culture measurements with other outcome measures in relation to patient safety improvement strategies over time [18]. Future research will also consider how information from measurements of patient safety culture is used by Swedish health care organizations and units.

Limitations to the findings of our study are at least twofold. First, the sample used for validation of psychometric properties was a subset of the whole sample with all items answered. It has been shown that responders who have all items answered are mainly those with direct patient interaction [13]. Thus, the factor analyses may mainly build on responses from staff working with direct patient contact and the material not being representative for all staff members. Also, primary care responders may more often leave questions unanswered if the item is not relevant to them. Secondly, proving correlation between an within-method outcome measure like self-estimated grade and the dimensions of the instrument has been questioned [9].

## Conclusions

The Swedish version of HSOPSC, the S-HSOPSC, consisting of 14 dimensions, 48 items and 3 single-item outcome measures, is widely used both in hospitals and in primary care settings. The assessment of its construct validity and internal consistency in a large dataset of first-time responders which is reported in this paper showed acceptable results both for the total sample and for the two subsamples: the hospital and primary care samples. This study suggests that the instrument can be used in both hospital and primary care settings after minor adjustments of wordings. There are advantages to one common instrument for measurements of patient safety culture as it allows comparisons within the health care system and assessments of national patient safety improvement programs. The S-HSOPSC needs to be validated as a performance measurement tool by comparing the results from safety culture measurements with other outcome measurements over time and confirming its usefulness as a tool for patient safety improvement work in Swedish health care.

## Abbreviations

AHRQ: Agency for Healthcare Research and Quality; HSOPSC: Hospital Survey on Patient Safety Culture; S-HSOPSC: Swedish version of Hospital Survey on Patient Safety Culture; EFA: Exploratory factor analysis; CFA: Confirmatory factor analysis; CFI: Comparative fit index; GFI: Goodness of fit index; AGFI: Adjusted goodness of fit index; NFI: Normalized fit index; NNFI: Non-normalized fit index; RMSEA: Root mean square error of approximation; AVE: Average variance extracted; CR: Construct reliability.

## Competing interests

The authors declare that they have no competing interests.

## Authors’ contributions

MH, KPH and MAS designed the study and wrote the manuscript. ML and MS have been currently involved in evaluating the results and the decision making concerning use of and changes to the instrument and given valuable contributions to the manuscript. MH and EB have done the statistical analyses and EB has also contributed to the manuscript. JØ has given valuable advice to the design of the study and has critically revised the manuscript. All authors read and approved the final manuscript.

## Pre-publication history

The pre-publication history for this paper can be accessed here:

http://www.biomedcentral.com/1472-6963/13/332/prepub

## Supplementary Material

Additional file 1: Appendix 1 Explorative Factor Analysis (EFA) of the Swedish version of Hospital Survey on Patient Safety Culture (S-HSOPSC). Click here for file
