# Supplementary material for: Psychometric properties of the hospital survey on patient safety culture, HSOPSC, applied on a large Swedish health care sample
Source: BMC Health Serv Res. 2013 Aug 22;13:332. doi: 10.1186/1472-6963-13-332 (PMC3765335; doi:10.1186/1472-6963-13-332)
Supplement: Additional file 1: Appendix 1 — Explorative Factor Analysis (EFA) of the Swedish version of Hospital Survey on Patient Safety Culture (S-HSOPSC). [file 1472-6963-13-332-S1.doc]

| **Total Sample Pattern Matrix^a^** | | | | | | | | | | **Hospital Care Sample Pattern Matrix^a^** | | | | | | | | | **Primary Care Sample Pattern Matrix^a^** | | | | | | | | |
| --- | --- | --- | --- | --- | --- | --- | --- | --- | --- | --- | --- | --- | --- | --- | --- | --- | --- | --- | --- | --- | --- | --- | --- | --- | --- | --- | --- |
|  | Factor | | | | | | | | | Factor | | | | | | | | | Factor | | | | | | | | |
|  | 1 | 2 | 3 | 4 | 5 | 6 | 7 | 8 | 9 | 1 | 2 | 3 | 4 | 5 | 6 | 7 | 8 | 9 | 1 | 2 | 3 | 4 | 5 | 6 | 7 | 8 | 9 |
| **1** | **Communication openness** | | | | | | | | |  |  |  |  |  |  |  |  |  |  |  |  |  |  |  |  |  |  |
| C2 | ,486 |  |  |  |  |  |  |  |  |  | ,500 |  |  |  |  |  |  |  |  |  | ,469 |  |  |  |  |  |  |
| C4 | ,302 |  |  |  |  |  |  |  |  |  | ,293 |  |  |  |  |  |  |  |  |  | ,258 |  |  |  |  |  |  |
| C6r |  |  |  |  |  |  |  | ,362 |  |  |  |  |  |  |  |  |  | ,311 |  |  |  |  |  |  | ,359 |  |  |
| **2** | **Feedback and communication about error** | | | | | | | | |  |  |  |  |  |  |  |  |  |  |  |  |  |  |  |  |  |  |
| C1 | ,738 |  |  |  |  |  |  |  |  |  | ,777 |  |  |  |  |  |  |  |  |  | ,728 |  |  |  |  |  |  |
| C3 | ,848 |  |  |  |  |  |  |  |  |  | ,885 |  |  |  |  |  |  |  |  |  | ,790 |  |  |  |  |  |  |
| C5 | ,679 |  |  |  |  |  |  |  |  |  | ,698 |  |  |  |  |  |  |  |  |  | ,773 |  |  |  |  |  |  |
| **3** | **Frequency of error reporting** | | | | | | | | |  |  |  |  |  |  |  |  |  |  |  |  |  |  |  |  |  |  |
| D1 |  |  |  |  |  |  | ,839 |  |  |  |  |  |  |  | ,838 |  |  |  |  |  |  |  |  | ,840 |  |  |  |
| D2 |  |  |  |  |  |  | ,930 |  |  |  |  |  |  |  | ,925 |  |  |  |  |  |  |  |  | ,901 |  |  |  |
| D3 |  |  |  |  |  |  | ,682 |  |  |  |  |  |  |  | ,679 |  |  |  |  |  |  |  |  | ,674 |  |  |  |
| **4** | **Handoffs & transitions between units and shifts** | | | | | | | | |  |  |  |  |  |  |  |  |  |  |  |  |  |  |  |  |  |  |
| F3r |  |  |  | ,529 |  |  |  |  |  |  |  |  | ,668 |  |  |  |  |  | ,777 |  |  |  |  |  |  |  |  |
| F5r |  |  |  | ,694 |  |  |  |  |  |  |  |  | ,620 |  |  |  |  |  |  |  |  |  |  |  |  |  | ,632 |
| F7r |  |  |  | ,705 |  |  |  |  |  |  |  |  | ,791 |  |  |  |  |  | ,661 |  |  |  |  |  |  |  |  |
| F11r |  |  |  | ,593 |  |  |  |  |  |  |  |  | ,500 |  |  |  |  |  |  |  |  |  |  |  |  |  | ,577 |
| **5** | **Executive management support for patient safety** | | | | | | | | |  |  |  |  |  |  |  |  |  |  |  |  |  |  |  |  |  |  |
| F1 |  |  |  |  | ,708 |  |  |  |  |  |  |  |  |  |  | ,681 |  |  | ,518 |  |  |  |  |  |  |  |  |
| F8 |  |  |  |  | ,785 |  |  |  |  |  |  |  |  |  |  | ,735 |  |  | ,577 |  |  |  |  |  |  |  |  |
| F9r |  |  |  |  | ,573 |  |  |  |  |  |  |  |  |  |  | ,535 |  |  | ,449 |  |  |  |  |  |  |  |  |
| **6** | **Non punitive response to error** | | | | | | | | |  |  |  |  |  |  |  |  |  |  |  |  |  |  |  |  |  |  |
| A8r |  |  |  |  |  |  |  | ,569 |  |  |  |  |  |  |  |  |  | ,588 |  |  |  |  |  |  | ,611 |  |  |
| A12r |  |  |  |  |  |  |  | ,595 |  |  |  |  |  |  |  |  |  | ,602 |  |  |  |  |  |  | ,593 |  |  |
| A16r |  |  |  |  |  |  |  | ,573 |  |  |  |  |  |  |  |  |  | ,581 |  |  |  |  |  |  | ,550 |  |  |
| **7** | **Organizational learning – continuous improvement** | | | | | | | | |  |  |  |  |  |  |  |  |  |  |  |  |  |  |  |  |  |  |
| A6 | ,478 |  |  |  |  |  |  |  |  |  | ,453 |  |  |  |  |  |  |  |  |  | ,568 |  |  |  |  |  |  |
| A9 | ,426 |  |  |  |  |  |  |  |  |  | ,426 |  |  |  |  |  |  |  |  |  | ,429 |  |  |  |  |  |  |
| A13 | ,402 |  |  |  |  |  |  |  |  |  | ,406 |  |  |  |  |  |  |  |  |  | ,496 |  |  |  |  |  |  |
| **8** | **Overall perception of safety** | | | | | | | | |  |  |  |  |  |  |  |  |  |  |  |  |  |  |  |  |  |  |
| A15 |  | ,263 |  |  |  |  |  |  |  | ,296 |  |  |  |  |  |  |  |  |  |  |  | ,206 |  |  |  |  |  |
| A18 | ,390 |  |  |  |  |  |  |  |  |  | ,374 |  |  |  |  |  |  |  |  |  | ,490 |  |  |  |  |  |  |
| A10r |  | ,582 |  |  |  |  |  |  |  | ,643 |  |  |  |  |  |  |  |  |  |  |  | ,524 |  |  |  |  |  |
| A17r |  | ,521 |  |  |  |  |  |  |  | ,579 |  |  |  |  |  |  |  |  |  |  |  | ,467 |  |  |  |  |  |
| **9** | **Staffing** | | | | | | | | |  |  |  |  |  |  |  |  |  |  |  |  |  |  |  |  |  |  |
| A2 |  | ,634 |  |  |  |  |  |  |  | ,667 |  |  |  |  |  |  |  |  |  |  |  | ,620 |  |  |  |  |  |
| A5r |  | ,486 |  |  |  |  |  |  |  | ,471 |  |  |  |  |  |  |  |  |  |  |  | ,491 |  |  |  |  |  |
| A7r |  | ,369 |  |  |  |  |  |  |  | ,369 |  |  |  |  |  |  |  |  |  |  |  | ,396 |  |  |  |  |  |
| A14r |  | ,737 |  |  |  |  |  |  |  | ,741 |  |  |  |  |  |  |  |  |  |  |  | ,745 |  |  |  |  |  |
| **10** | **Supervisor/manager expectations & actions promoting safety** | | | | | | | | | |  |  |  |  |  |  |  |  |  |  |  |  |  |  |  |  |  |
| B1 |  |  |  |  |  |  |  |  | ,735 |  |  |  |  |  |  |  | ,719 |  |  |  |  |  |  |  |  | ,668 |  |
| B2 |  |  |  |  |  |  |  |  | ,832 |  |  |  |  |  |  |  | ,846 |  |  |  |  |  |  |  |  | ,660 |  |
| B3r |  |  |  |  |  |  |  |  | ,460 |  |  |  |  |  |  |  | ,504 |  |  |  |  |  |  |  |  | ,428 |  |
| B4r |  |  |  |  |  |  |  |  | ,565 |  |  |  |  |  |  |  | ,615 |  |  |  |  |  |  |  |  | ,455 |  |
| **11** | **Teamwork across units** | | | | | | | | |  |  |  |  |  |  |  |  |  |  |  |  |  |  |  |  |  |  |
| F4 |  |  |  |  | ,410 |  |  |  |  |  |  |  | ,446 |  |  |  |  |  | ,740 |  |  |  |  |  |  |  |  |
| F10 |  |  |  |  | ,392 |  |  |  |  |  |  |  | ,455 |  |  |  |  |  | ,674 |  |  |  |  |  |  |  |  |
| F2r |  |  |  |  | ,476 |  |  |  |  |  |  |  | ,504 |  |  |  |  |  | ,685 |  |  |  |  |  |  |  |  |
| F6r |  |  |  | ,455 |  |  |  |  |  |  |  |  | ,442 |  |  |  |  |  |  |  |  |  |  |  |  |  | ,471 |
| **12** | **Teamwork within the unit** | | | | | | | | |  |  |  |  |  |  |  |  |  |  |  |  |  |  |  |  |  |  |
| A1 |  |  |  |  |  | ,763 |  |  |  |  |  |  |  | ,820 |  |  |  |  |  |  |  |  | ,657 |  |  |  |  |
| A3 |  |  |  |  |  | ,693 |  |  |  |  |  |  |  | ,648 |  |  |  |  |  |  |  |  | ,718 |  |  |  |  |
| A4 |  |  |  |  |  | ,702 |  |  |  |  |  |  |  | ,773 |  |  |  |  |  |  |  |  | ,609 |  |  |  |  |
| A11 |  |  |  |  |  | ,516 |  |  |  |  |  |  |  | ,464 |  |  |  |  |  |  |  |  | ,607 |  |  |  |  |
| **13** | **Information and support to patients & family who have suffered an adverse event** | | | | | | | | | | | | |  |  |  |  |  |  |  |  |  |  |  |  |  |  |
| G3 |  |  | ,761 |  |  |  |  |  |  |  |  | ,770 |  |  |  |  |  |  |  | ,745 |  |  |  |  |  |  |  |
| G4 |  |  | ,880 |  |  |  |  |  |  |  |  | ,880 |  |  |  |  |  |  |  | ,871 |  |  |  |  |  |  |  |
| G5 |  |  | ,831 |  |  |  |  |  |  |  |  | ,829 |  |  |  |  |  |  |  | ,833 |  |  |  |  |  |  |  |
| G6 |  |  | ,662 |  |  |  |  |  |  |  |  | ,678 |  |  |  |  |  |  |  | ,686 |  |  |  |  |  |  |  |
| **14** | **Information and support to staff who have been involved in an adverse event** | | | | | | | | | | | | |  |  |  |  |  |  |  |  |  |  |  |  |  |  |
| G7 |  |  | ,451 |  |  |  |  |  |  |  |  | ,438 |  |  |  |  |  |  |  | ,544 |  |  |  |  |  |  |  |
| G8 |  |  | ,407 |  |  |  |  |  |  |  |  | ,384 |  |  |  |  |  |  |  | ,465 |  |  |  |  |  |  |  |

Rotation Method: Promax with Kaiser Normalization.

Total sample

1. 9 factors extracted. 11 iterations required.

Hospital sample

1. 9 factors extracted. 11 iterations required.

Total sample

1. 9 factors extracted. 10 iterations required.
